# Myofascial trigger points in migraine and tension-type headache

**DOI:** 10.1186/s10194-018-0913-8

**Published:** 2018-09-10

**Authors:** Thien Phu Do, Gerda Ferja Heldarskard, Lærke Tørring Kolding, Jeppe Hvedstrup, Henrik Winther Schytz

**Affiliations:** Headache Diagnostic Laboratory, Danish Headache Center and Department of Neurology, Rigshospitalet Glostrup, Faculty of Health Sciences, University of Copenhagen, Glostrup, Denmark

**Keywords:** Headache, Myofascial trigger point, Muscle, Treatment, Trigemino-cervical-complex, Migraine, Tension-type headache, Diagnostic test

## Abstract

**Background:**

A myofascial trigger point is defined as a hyperirritable spot in skeletal muscle that is associated with a hypersensitive palpable nodule in a taut band. It has been suggested that myofascial trigger points take part in chronic pain conditions including primary headache disorders. The aim of this narrative review is to present an overview of the current imaging modalities used for the detection of myofascial trigger points and to review studies of myofascial trigger points in migraine and tension-type headache.

**Findings:**

Different modalities have been used to assess myofascial trigger points including ultrasound, microdialysis, electromyography, infrared thermography, and magnetic resonance imaging. Ultrasound is the most promising of these modalities and may be used to identify MTrPs if specific methods are used, but there is no precise description of a gold standard using these techniques, and they have yet to be evaluated in headache patients.

Active myofascial trigger points are prevalent in migraine patients. Manual palpation can trigger migraine attacks. All intervention studies aiming at trigger points are positive, but this needs to be further verified in placebo-controlled environments. These findings may imply a causal bottom-up association, but studies of migraine patients with comorbid fibromyalgia syndrome suggest otherwise. Whether myofascial trigger points contribute to an increased migraine burden in terms of frequency and intensity is unclear.

Active myofascial trigger points are prevalent in tension-type headache coherent with the hypothesis that peripheral mechanisms are involved in the pathophysiology of this headache disorder. Active myofascial trigger points in pericranial muscles in tension-type headache patients are correlated with generalized lower pain pressure thresholds indicating they may contribute to a central sensitization. However, the number of active myofascial trigger points is higher in adults compared with adolescents regardless of no significant association with headache parameters. This suggests myofascial trigger points are accumulated over time as a consequence of TTH rather than contributing to the pathophysiology.

**Conclusions:**

Myofascial trigger points are prevalent in both migraine and tension-type headache, but the role they play in the pathophysiology of each disorder and to which degree is unclarified. In the future, ultrasound elastography may be an acceptable diagnostic test.

## Background

Migraine affects 16% of the population in Europe [1] with high individual and socioeconomic costs [2, 3]. Several mechanisms have been proposed to be involved in its pathophysiology including vascular, peripheral and central mechanisms [4–9]. Jes Olesen systematically described pericranial tenderness in migraine patients, both during and outside of migraine attacks [10, 11], leading to speculations that myofascial mechanisms may be involved in migraine [12].

Tension-type headache (TTH) is the most prevalent primary headache disorder worldwide [13]. Tenderness in pericranial myofascial tissue is correlated with the intensity and frequency of headache in TTH [14–16], and studies show increased muscle stiffness in TTH patients [17, 18]. Thus, myofascial structures may be associated with TTH pathophysiology.

The term myofascial trigger point (MTrP) was popularized in the 1950s and is defined as a hyperirritable spot in skeletal muscle that is associated with a hypersensitive palpable nodule in a taut band [19, 20]. The spot is painful on compression and can cause referred pain, referred tenderness, motor dysfunction and autonomic phenomena. The interest in myofascial symptoms has been ongoing for centuries with similar descriptions of localized thickenings of muscles with regional pain [21]. There have been inconsistencies and controversies in the literature on the underlying pathology, and even the existence of MTrPs [22]. While attempts have been made to visualize MTrPs [22], the gold standard for detection of MTrPs has been unchanged since the 1950s [22] and remains to be by way of palpation of the affected muscles. However, this technique proves to be poorly reproducible as practitioners disagree on the location of MTrPs when blindly examining different patient groups [23]. Nevertheless, MTrPs have come to play a central role in the diagnosis and treatment of myofascial pain syndrome [19]. Furthermore, MTrPs have been proposed to take part in primary headache disorders and other chronic pain conditions [12]. The aim of this narrative review is to present an up-to-date overview on MTrPs in general and then in migraine and TTH, respectively.

## Review

### Myofascial trigger points

In the comprehensive trigger point manual by Travell and Simons [19], MTrPs are subclassified into different types, e.g., active and latent amongst others. An active MTrP produces a constant pain complaint while a latent only produces pain during manual palpation [19]. It was hypothesized that a sustained muscle contraction in MTrPs promotes hypoxia and ischemia with a following increase in concentrations of substances such as calcitonin gene-related peptide (CGRP) and substance P (SP) [24]. Consequently, this would lead to increased peripheral nociceptive transmission [24]. This hypothesis is only supported in active MTrPs, as they have been shown to be associated with higher levels of these substances in the local milieu compared to latent MTrPs [25, 26]. Other properties such as the consistency of the tissue have also been suggested to play a key role in MTrPs [27].

### Investigations of myofascial trigger points

#### Ultrasound imaging

Different ultrasound modalities in ultrasound imaging have visualized MTrPs. Lewis et al. [28] conducted a pilot study to assess the use of ultrasound in determining soft tissue changes in the region of active MTrPs in 11 subjects. They found no correlation between clinical identified active MTrPs and ultrasound. In contrast, Turo et al. [29] were able to differentiate between symptomatic MTrPs and asymptomatic muscle tissue with texture-based analysis. Sikdar et al. investigated the stiffness of active and latent MTrPs, using ultrasound elastography by Doppler variance imaging in nine subjects while inducing vibrations with an external handheld massage vibrator [27]. MTrPs appeared as focal, hypoechoic regions on two-dimensional ultrasound images and with reduced vibration amplitude, indicating increased stiffness. Furthermore, they describe hypoechoic regions that were not identified during palpation prior to ultrasound. In another study by the same group, MTrPs showed reduced vibration amplitude on elastography indicating increased stiffness and distinct blood flow waveform patterns [30]. Ballyns et al. [31] used elastography to investigate MTrPs in 44 subjects with acute cervical pain. They were able to measure the size and distinguish type (active, latent) of MTrPs with elastography. In addition, Doppler waveforms of blood flow showed different characteristics in active sites compared to normal tissue. Takla et al. [32] compared elastography with two-dimensional grayscale ultrasound in identifying MTrPs. They found that MTrPs had an accuracy of 100% for both active and latent MTrPs while two-dimensional grayscale ultrasound could only identify 33 and 35%, respectively.

#### Microdialysis

Microdialysis has been used to measure endogenous and exogenous molecules in the local milieu of MTrPs. Shah et al. [25] used microdialysis to investigate subjects with active or latent MTrP, and controls without MTrP were detected by manual palpation by two experienced clinicians. The authors measured selected substances (pH, bradykinin (BK), CGRP, SP, tumor necrosis factor alpha (TNF-α), interleukin 1 beta (IL-1β), interleukin 6 (IL-6), interleukin 8 (IL-8), serotonin, and norepinephrine (NE)) in standardized locations of the trapezius muscle and gastrocnemius muscle. Subjects with active MTrPs in the trapezius muscle showed increased concentrations of all substances compared to the other groups. Shah et al. [26] found similar results in the trapezius muscle of subjects with neck pain and active MTrP compared to a group with neck pain and no MTrP present and healthy controls. The results showed that the active MTrP group had higher concentrations of BK, CGRP, SP, TNF-α, IL-1β, serotonin, NE.

#### Electromyography

Electromyography (EMG) can be used to measure the electrical activity of skeletal muscles. Simons et al. compared the prevalence of motor endplate potentials in active MTrPs, endplate zones, and taut bands of skeletal muscles in subjects with palpable MTrPs [33]. The authors found that endplate noise was more common in MTrPs than in sites outside of the trigger point, even within the same endplate zone. Ge et al. evaluated intramuscular muscle activity in a synergistic muscle during isometric contraction in 15 subjects with latent MTrPs [34]. The needle was inserted into a latent MTrP or a non-MTrP in the upper trapezius at rest and during contraction. The EMG activities were recorded from the middle deltoid muscle and the upper, middle, and lower parts of the trapezius muscle. The intramuscular EMG activity in the upper trapezius muscle was significantly higher at rest and during contraction at latent MTrPs compared with non-MTrPs. Yu et al. measured maximum voluntary isometric contraction, endurance, median frequency, and muscle fatigue index in three groups of participants: an active MTrP group, a latent MTrP group, and a control group [35]. The active MTrP group had a higher median frequency and muscle fatigue index than the control group. Wytrążek et al. compared the EMG activity of muscle motor units at rest and maximal contraction with surface EMG recordings [36]. The results showed MTrPs correlated with an increase in EMG amplitude at rest.

#### Infrared thermography

Infrared thermography can be used to measure the skin temperature. Dibai-Filho et al. [37] have reviewed the literature on infrared thermography investigations of MTrPs. The authors included three comparative studies [38–40] and one accuracy study [41]. The conclusion of the review is that the included studies do not agree on skin temperature patterns in the presence of MTrPs. The included studies of the review are briefly presented in the following. Merla et al. [38] found that individuals with myofascial pain had a greater difference between the right and left side in skin temperature over the masseter and sternocleidomastoid muscles before and after maximal voluntary clenching compared to healthy volunteers. They also found that the myofascial pain group had a greater temperature change over the measured muscles after maximum voluntary clenching. Kimura et al. [39] evaluated the vasoconstrictor response after provoking pain in MTrPs with an intramuscular glutamate injection. Furthermore, they activated the sympathetic outflow by using a breath-holding maneuver. They found a decrease in skin temperature over time in latent MTrPs. In contrast, Zhang et al. [40] did not find that the skin temperature was affected following an intramuscular glutamate injection into latent MTrPs. Haddad et al. [41] compared infrared thermography and algometer measurements of MTrPs in the masticatory muscles. The authors found a positive correlation between skin surface temperature and pressure pain threshold. Regarding diagnosing MTrPs, infrared thermography had an accuracy of 0.564 to 0.609 (area under the receiver operating characteristic curve).

#### Magnetic resonance imaging

Chen et al. [42] examined 65 patients with myofascial pain-associated taut bands using magnetic resonance elastography. They found that agreement between physicians and imaging raters were relatively poor (63%; 95% CI, 50%–75%), but that these bands could be assessed quantitatively using magnetic resonance elastography. The authors suggest that clinicians might overestimate while magnetic resonance elastography may underestimate MTrPs.

### Migraine and myofascial trigger points

Pericranial tenderness in migraine patients was systematically described by Jes Olesen in 1978, both during and outside of attacks [10, 11] leading to speculations that myofascial mechanisms may be involved in migraine [12]. The bottom-up model states that increased peripheral nociceptive transmission sensitizes the central nervous system to lower the threshold for perceiving pain while the top-down model suggests these changes are already present in the central nervous system [43]. While it can be argued that pericranial tenderness in migraine may be caused by a top-down central sensitization, a bottom-up association was implied in 1981 when Tfelt-Hansen et al. [44] demonstrated that injections of lidocaine and saline into tender trigger points could relieve migraine attacks. They infiltrated the most tender spots of 26 cranial and neck muscles and tendon insertions in 50 migraine patients. The most frequent sites of tenderness were sternocleidomastoid, anterior temporal, neck and shoulder muscles, the coronoid process and occipital insertions. The tender points in the mentioned study do not necessarily overlap with Travell and Simons’ definition of MTrPs, but the implication stands that peripheral myofascial mechanisms may be involved in migraine pathophysiology. Consequently, there has been an interest in exploring MTrPs in migraine (Table 1) [45–58].

**Table 1:** Migraine and myofascial trigger points

First author (year)	Blinding	Participants	Mean age (range)	Gender	Timing of recordings	Methods	Muscles	Main findings
Calandre (2006) [45]	None	8 EMA 55 EMO 35 CMO 32 CTRLs (18 (56%) of these reported infrequent TTH)	38.5 ± 13.5 (15–75) 41.4 ± 16.8 (21–83)	9 M, 79F 13 M, 19F	Interictally	MTrP diagnosis by manual palpation with a pressure by no more than 4 kg. The number and location of trigger points in each patient were recorded.	Frontal, temporal, and superior trapezius muscles and suboccipital and occipital area	• 93.9% migraine patients reported referred pain. • The number of MTrPs correlated with frequency and duration of migraine attacks.
Fernández-de-Las-Peñas (2006) [46]	Examiner blinded to diagnosis	5 EMA 15 EMO 20 CTRLs	33 ± 10 (17–57) 30 ± 8 (19–55)	7 M, 13F 8 M, 12F	Interictally	MTrP diagnosis was performed following the criteria described by Simons et al. [19] and by Gerwin et al. [89] FHP was documented in relaxed standing position and relaxed sitting position. Neck mobility was assessed.	Upper trapezius, sternocleidomastoid, temporalis, and subocciptal muscles	• Active MTrPs were only found in the migraine patients. • Active MTrPs were primarily located ipsilateral to the migraine headaches except for the suboccipital region. • Migraine patients have a greater FHP and less neck motility in extension and flexion-extension compared to controls.
Ferracini (2017) [47]	Examiner blinded to diagnosis	98 EM 45 CM With or without aura not reported.	37 ± 12 (18–60) 38 ± 12 (18–60)	143F	Interictally	MTrP diagnosis was performed following the criteria described by Simons et al. [19] and by Gerwin et al. [89] The Migraine Disability Assessment Scale (MIDAS) questionnaire was used.	Temporalis, masseter, suboccipital, sternocleidomastoid, upper trapezius and splenius capitis	• No significant difference was in the total number of MTrPs between the two groups. • Active MTrPs in the temporalis and masseter muscle were most prevalent in both groups. • The number of MTrPs did not correlate with migraine related disability nor migraine features.
Ferracini (2016) [48]	None	50 EM With or without aura not reported.	34.1 (18–55)	5 M, 45F	Interictally: 46% Ictally: 54%	MTrP diagnosis was performed following the criteria described by Simons et al. [19] and by Gerwin et al. [89] Eight measures of head and neck posture were obtained by radiograph and different angles were defined.	Temporalis, masseter, suboccipital, sternocleidomastoid,, upper trapezius, and splenius capitis	• Individuals with migraine showed MTrPs in all the muscles. • Active MTrPs was positively associated with a reduction in cervical lordosis and head extension of the head on the neck. • No association between the number of active MTrPs and clinical features of migraine was observed.
Florencio (2017) [49]	None	70 EMO	42 ± 12 (39–45)	70F	Interictally	MTrP diagnosis was performed following the criteria described by Simons et al. [19] and by Gerwin et al. [89] Surface EMG was recorded from superficial flexor and extensor muscles bilaterally as subjects performed a staged task of cranio-cervical flexion. The average Root Mean Square (RMS) was calculated from each 10 s contraction.	Sternocleidomastoid, upper trapezius and splenius capitis	• All patients exhibited active MTrPs in their cervical muscles • Participants with active MTrPs in the included muscles had lower normalized RMS in their superficial neck flexors • Subjects with active MTrPs in the splenius capitis and upper trapezius had higher normalized RMS values in the splenius capitis.
Gandolfi (2017) [50]	Single-blind	22 CM patients receiving onabotulinumtoxinA treatment Patients were divided into two groups: 12 individuals receiving manipulative treatment 10 individuals receiving electrical stimulation (placebo group) With or without aura not reported.	45.8 ± 14.1 (18–66) 50.2 ± 6.2 (40–61)	3 M, 19F 2 M, 10F 1 M, 9F	Not reported	Patients were randomly assigned to receive either manipulative treatment (treatment aimed at improving mobility and reducing stiffness in the cervicothoracic spine) or transcutaneous electrical nerve stimulation in the upper trapezius. Treatment consisted of 4 sessions (30 min once a week in 4 weeks) Patients were asked to keep a headache diary: outcomes were evaluated before treatment, during treatment, and 1 month after the end of treatment. Cervical active range of motion and trigger point sensitivity were measured pre- and posttreatment. MTrP sensitivity was assessed by measuring PPT using an algometer.	Frontalis, temporalis, occipital, and trapezius	• The total consumption of analgesics and NSAIDs was significantly lower in the patients treated with manipulative treatment than in those treated with electrical stimulation. • The PPTs at the MTrPs in the upper trapezius, occipital and temporal muscles were significantly lower in the patients treated with manipulative treatment than in those treated with electrical stimulation. • After trial patients who received manipulative treatment had a significantly lower consumption of NSAIDs, analgesics and triptans.
Ghanbari (2015) [51]	None	44 migraine patients Whether patients had chronic or episodic migraine with or without aura was not reported.	37.25 38.63 35.86 Range not reported	20 M, 24F 9 M, 13F 11 M, 11F	Not reported	MTrPs were considered to be active if 1) referred pain due to palpation reproduced the subjects’ headache. 2) There was a jump sign that was the characteristic behavioral response to pressure on a trigger point. All subjects included had active trigger points. Subjects (al were randomly assigned to one of two groups: 1) Medication only 2) Medication + positional release therapy The treatment phase lasted 2 weeks and medication included NSAIDs, nortriptyline, propranolol and depakine. Subjects completed a daily headache diary throughout the study and tablet count was recorded. After a baseline period of 2 weeks the sensitivity of trigger points (using a digital force gauge) and cervical range of motion were assessed. This was repeated after the treatment phase and as a follow up after 1, 2 and 4 months (counting from start of treatment)	Suboccipital, sternocleidomastoid, upper trapezius, cervical multifidus, rotators and interspinales	• Both groups showed significant reduction in headache intensity, frequency, duration and tablet count after 4 months follow up. • The sensitivity of trigger points was significantly reduced in the medication positional release therapy group, while it remained unchanged in the medicine only group.
Giamberardino (2007) [52]	Examiner blinded to diagnosis	Primary experiment 78 MO (7 also diagnosed with TTH) 20 healthy CTRLs Secondary experiment 12 MO (2 also diagnosed with TTH)	31.4 ± 5.8 (23–46) 33.3 ± 7 (18–46) 29.3 ± 4 (24–35) 32.33 ± 6.44 (24–44)	11 M, 43F 5 M, 19F 5 M, 15F 3 M, 9F	Interictally	MTrP diagnosis was performed following the criteria described by Simons et al. [19] A MTrP was considered active if palpation induced both local and referred pain. Pain threshold was assessed by electrical stimulation. Subsequent to threshold measurements group 1 also received 0,5 mL bupivacaine (5 mg/mL). The infiltration and pain threshold measurements were repeated on the 3., 10., 30., and 60. day. PPT in healthy controls was assessed with the same frequency. Migraines (number and intensity of attacks) were assessed 60 days prior to the study and 60 days after the study started. This was done using a headache diary. The second study is a 30 days “placebo-like study” where saline was injected near the MTrPs. PPT, injections and the headache diary were fulfilled similarly to the the prior experiment. (only up till 30 days)	Sternocleidomastoid, semispinalis cervicis, splenius cervicis	• Group 1 and 2 pain thresholds were significantly lower than in controls at baseline. In group one pain threshold increased significantly during treatment. In group two there was no significant change. In the control group there was no significant variation. • In group 1 maximal intensity and number of migraine attacks decreased significantly. In group 2 the change was not significant. • The mean number of rescue medication taken fell significantly in group 1, but not in group 2. • The group that participated in the second experiment also had a pain threshold lower than normal.
Landgraf (2017) [53]	None	26 adolescent migraine patients (chronic/episodic not reported) 17 MO 5 MA 4 with vestibular migraine	14.5 (6.3–17.8)	13 M, 13F	Not specified	MTrPs were identified by palpation and the PPT on these points was measured using an algometer. Manual pressure was applied to the trigger points, and the occurrence and duration of induced headache were recorded. At a second consultation (4 weeks after the first), manual pressure with the detected pressure threshold was applied to non-trigger points within the same trapezius muscle (control).	Trapezius muscle	• Manual pressure to MTrPs in the trapezius muscle led to lasting headache after termination of the manual pressure in 13 (50%) patients (from 5 s to over 30 min). • No patient experienced headache when manual pressure was applied to non-trigger points at the control visit. • Headache was induced significantly more often in children ≥12 years and those with internalizing behavioral disorder.
Landgraf (2015) [54]	None	3 migraine patients Whether patients had chronic or episodic migraine with or without aura was not reported.	23.67 (23–24)	1 M, 2F	Interictally	MTrP diagnosis was performed following the criteria described by Simons et al. [19] and by Gerwin et al. [89] These areas were marked by nitroglycerin capsules on the adjacent skin surface. High-resolution MR imaging of the posterior cervico-cranial muscles was performed on a 3 T MR scanner with a spine array as well as surface coils. High resolution T2 weighted and T1-weighted sequences as well as short tau inversion recovery (STIR) sequences were acquired in a coronaland axial slice orientation.	Trapezius	• MR imaging demonstrated focal, partly T2 hyper intense signal alterations within the trapezius muscles in all three study participants. All of the observed signal alterations were in close proximity to the fiducial markers taped on the skin.
Palacios-Ceña (2017) [55]	None	95 EM With or without aura not reported.	40 (37–43)	0 M, 95F	Interictally	MTrP diagnosis was performed following the criteria described by Simons et al. [19]. PPT was assessed using an algometer.in the following regions: • Over the temporalis muscle. • C5/C6 zygapophyseal joint. • Tibialis anterior muscle (a pain-free distant control site)	Temporalis, masseter, suboccipital, sternocleidomastoid, upper trapezius, and splenius capitis	• The higher the intensity of migraine pain, the lower the PPTs over the cervical spine. • The number of active MTrPs was significantly and negatively associated with PPT in all the points.
Ranoux (2017) [56]	None	7 CMA 50 CMO 57 chronic migraine patients (refractory to conventional treatment)	44.3 (17–85)	14 M, 43F	Not specified	Observational, open label, real-life, cohort study. The patients were injected with OnabotulinumtoxinA using a “follow-the-pain” pattern in MTrPs.	Corrugator supercilii, temporalis and trapezius muscles	• 65.1% responded to treatment. • The associated cervical pain and muscle tenderness, present in 33 patients, was reduced by ≥50% in 31 patients (94%). • Triptan consumption decreased (81%) in responders.
Sollmann (2016) [57]	None	6 MO 14 MA (50% also had some degree of TTH) chronic/episodic not reported	23 ± 1.8 (19–27)	1 M, 19F	Interictally	rPMS (repetitive peripheral magnetic stimulation) was used to stimulate active MTrPs of the upper trapezius muscles. This was done in 6 stimulation sessions over 2 consecutive weeks. PPT was assessed using an algometer. Participants completed a standardized headache questionnaire including occurrence, duration and intensity of headaches. This was repeated over 3 months.	Trapezius and deltoid (as a control)	• In 19 subjects MTrP algometry values were significantly higher immediately after magnetic stimulation. • PPT increased during the trial.
Tali (2014) [58]	Examiner blinded to diagnosis during upper cervical fact joint mobility/stiffness MTrP evaluation not blinded	20 EM 20 CTRLs Distribution of with/without aura not reported.	24.95 ± 1.79 (20–27) 25.65 ± 1.42 (23–28)	2 M, 18F 3 M, 17F	Interictally	MTrP diagnosis was performed following the criteria described by Simons et al. [19] and Gerwin et al. [89] Neck range of motion was assessed using a cervical range of motion instrument. FHP was noted in a seated position. Upper cervical facet joint mobility/stiffness was evaluated using a motion palpation technique.	Sternocleidomastoid and upper trapezius muscle	• Active MTrPs were only found in the migraine group. • Significant differences were found in neck range of motion measurements and FHP between the migraine and control groups.

C* chronic, E* episodic, *MA* migraine with aura, *MO* migraine without aura, *CTRLs* healthy controls, *F* female, *M* male, *MTrP* myofascial trigger point, *EMG* electromyography, *PPT* pressure pain threshold, *FHP* forward head posture, *VAS* visual analog scale, *NRS* numeric rating scale

#### The occurrence of myofascial trigger points in migraine

Several studies have demonstrated a high occurrence of active and latent MTrPs in migraine patients [45–49]. Studies show that there is a significantly higher prevalence of active MTrPs in migraine patients compared to healthy controls [45, 46, 58]. There are conflicting results in which muscles are the most affected [47, 48]. Fernández-de-Las-Peñas et al. [46] observed that active MTrPs were most prevalent ipsilateral to the migraine headaches. More unclear is whether the amount of MTrPs is correlated with the frequency and intensity of headache attacks. Calandre et al. [45] found a positive correlation between the number of MTrPs and frequency and duration of migraine attacks, whereas two studies by Ferracini et al. [47, 48] found no such correlation. Interestingly, Landgraf et al. [54] could visualize MTrPs on MR imaging as focal signal alterations in a small pilot study.

#### Neck mobility and specific muscles

There appears to be an association between neck mobility and MTrPs [46, 48, 49, 58]. Ferracini et al. [48] found that a higher number of active MTrPs was positively correlated with a reduction in cervical lordosis and head extension of the head on the neck. In addition, that lower cervical angles were correlated higher then the number of active MTrPs. Florencio et al. [49] hypothesized that active MTrPs in the cervical musculature alters the activity of the related muscles and that this would be reflected in EMG readings. They observed that the presence of active MTrPs in the cervical musculature had different activation in the neck flexor muscles compared to those without active MTrPs in the same muscles regardless of the presence of pain. Palacios-Ceña et al. [55] found that the number of active MTrPs in head, neck and shoulder muscles were associated with widespread pressure hypersensitivity in a migraine population.

#### Provocation and intervention studies

Two unblinded studies show that manual palpation of MTrPs can provoke a migraine attack [45, 53]. Calandre et al. provoked a migraine attack in one-third of a migraine population by palpating MTrPs [45]. Landgraf et al. provoked migraine headache by inducing pressure to MTrPs and could not replicate this by pressure to non-trigger points in the trapezius in an adolescent migraine population [53].

Interventions targeted at MTrPs show promising results [50–52, 56, 57], but the quality of studies varies greatly and lack placebo-control. Giambierardino et al. demonstrated that local anesthetic infiltration of MTrPs resulted in a reduction of migraine symptomatology in terms of frequency and intensity [52]. Furthermore, there was a reduction of hyperalgesia, not only at the injection site but also in referred areas overlapping with migraine pain sites. Similar, Ranoux et al. injected botulinum toxin in MTrPs with similar results in terms of reduction in headache days [56]. Gandolfi et al. improved the outcome of prophylactic botulinum toxin treatment in chronic migraine patients with manipulative treatment of MTrPs [50]. The outcome was a lower consumption of analgesics, improvement in pressure pain threshold and increased cervical range of motion. Likewise, Ghanbari et al. reported that combined positional release therapy targeted at MTrPs with medical therapy is more effective than the sole pharmacological treatment [51]. Interestingly, sessions of magnetic stimulation of active MTrPs reduced headache frequency and intensity in adolescent migraineurs [57]. Though these findings need to be verified in a placebo-controlled study. There has not been any studies on the effect of systemic musculoskeletal analgesics on MTrPs [59], which would be of interest for future studies.

### Tension-type headache and myofascial trigger points

Both peripheral and central mechanisms have been suggested as important components of TTH [14–16, 60]. Tenderness in pericranial myofascial tissue is correlated with the intensity and frequency of headache [14–16]. Furthermore, there has been demonstrated increased muscle stiffness in the trapezius muscle in TTH patients [17, 18] not differing between headache and non-headache days [18]. Although a recent study found no increased muscle stiffness in TTH patients, this may be due to the method used [61]. Studies show that the referred pain elicited by active MTrPs reproduce the headache pattern in TTH patients [62–66]. Accordingly, there has been an interest in investigating the occurrence of MTrPs in TTH (Table 2) [62–80].

**Table 2:** Tension-type headache and myofascial trigger points

First author (year)	Blinding	Participants	Mean age (range)	Gender	Timing of recordings	Methods	Muscles	Main findings
Alonso-Blanco (2011) [62]	None	20 CTTH adult patients 20 CTTH adolescent patients	41 (18–47) 8 (6–12)	10 M, 10F 10 M, 10F	Interictally	MTrP diagnosis as described by Simons et al. [19]	Temporalis, suboccipital, sternocleidomastoid, and upper trapezius	• The number of active MTrPs were higher in adults versus children. • Referred pain elicited from active MTrPs shared similar pain patterns as spontaneous CTTH in both groups. No significant association between the number of active MTrPs and headache parameters.
Couppé (2007) [67]	Double-blinded	20 CTTH patients 20 CTRLs	37.5 (33.3–41.6)	Not reported	Ictally	MTrP diagnosis as described by Simons et al. [19] EMG examination at a MTrP and a control point in the same subject.	Upper trapezius	• The number of active MTrPs were higher in patients versus controls • No difference in electromyographic activity between MTrPs versus control points.
Fernández-de-las-Peñas (2011) [63]	Examiner blinded to diagnosis	50 CTTH patients 50 CTRLs	8 (6–12)	14 M, 36F	Interictally	MTrP diagnosis as described by Simons et al. [19].	Temporalis, superior oblique, masseter, suboccipital, sternocleidomastoid, levator scapulae, and upper trapezius	• Active MTrPs were only found in patients. • In the CTTH patients, the number of active TrPs correlated with the duration of a headache attack. • The local and referred pains elicited from active MTrPs shared similar pain pattern as spontaneous CTTH.
Fernández-de-las-Peñas (2009) [68]	None	40 CTTH	40 (20–57)	40F	Interictally < 4 on a 11 NRS	MTrP diagnosis was performed following the criteria described by Simons et al. [19] and Gerwin et al. [89] PPT was assessed using an algometer.	Temporalis (9 landmarks total, 3 each respectively in the anterior, medial and posterior part)	• The analysis of variance did not detect significant differences in the referred pain pattern between active MTrPs. • The topographical pressure pain sensitivity maps showed the distinct distribution of the MTrPs indicated by locations with low PPTs.
Fernández-de-las-Peñas (2007) [69]	Examiner blinded to diagnosis	15 ETTH 15 CTRLs	39 ± 17 (20–70) 37 ± 12 (21–70)	3 M, 12F 4 M, 11F	Interictally	MTrP diagnosis as described by Simons et al. [19] and Gerwin et al. [89] FHP was noted both seated and standing.	Temporalis, sternocleidomastoid, and upper trapezius	• Active MTrPs in the affected muscles were only found within the ETTH group. • MTrPs were not related to any clinical variable concerning the intensity and the temporal profile of headache.
Fernández-de-las-Peñas (2007) [70]	Examiner blinded to diagnosis	20 CTTH 20 CTRLs	36 (18–56) 35 (20–56)	11 M, 9F 13 M, 7F	< 4 cm on a 10 cm VAS	MTrP diagnosis as described by Simons et al. [19] and by Gerwin et al. [89] PPT was assessed using an algometer.	Upper trapezius	• CTTH subjects with active MTrPs showed greater headache intensity, and duration than those with latent TrPs. • Patients with bilateral MTrPs reported a greater headache intensity and duration than those with unilateral TrPs. • CCTH subjects showed a decreased PPT compared to controls.
Fernández-de-las-Peñas (2007) [66]	Examiner blinded to diagnosis	30 CTTH 30 CTRLs	39 ± 16 (18–65) 39 ± 12 (19–65)	9 M, 21F 9 M, 21F	< 4 cm on a 10 cm VAS	MTrP diagnosis as described by Simons et al. [19] and by Gerwin et al. [89]	Temporalis	• Referred pain was evoked in 87 and 54% on the dominant and non-dominant sides in CTTH patients, which was significantly higher than in controls (10% vs. 17%, respectively). • CTTH patients with active MTrPs in either right or left temporalis muscle showed longer headache duration than those with latent MTrPs. • CTTH patients showed significantly lower pressure pain threshold when compared with controls.
Fernández-de-las-Peñas (2006) [71]	Examiner blinded to diagnosis	10 ETTH 10 CTRLs	35 ± 15 (18–66) 34 ± 13 (18–66)	2 M, 8F 3 M, 7F	Interictally	MTrP diagnosis as described by Simons et al. [19] and by Gerwin et al. [89]	Suboccipital	• In the ETTH group, 60% showed active MTrPs; 40% showed latent trigger points. In the ETTH group, headache intensity, frequency and duration did not differ depending on whether the MTrPs were active or latent.
Fernández-de-las-Peñas (2006) [72]	Examiner blinded to diagnosis	25 CTTH 25 CTRLs	40 ± 16 (18–72) 38 ± 9 (18–73)	8 M, 17F 9 M, 16F	< 4 cm on a 10 cm VAS	MTrP diagnosis was performed following the criteria described by Simons et al. [19] and by Gerwin et al. [89] FHP was noted both seated and standing.	Temporalis, sternocleidomastoid, and upper trapezius	• Active MTrPs were only found in CTTH patients. • There was significant association between the presence of active MTrPs and headache intensity and duration.
Fernández-de-las-Peñas (2006) [65]	Examiner blinded to diagnosis	20 CTTH 20 CTRLs	38 ± 18 (18–70) 35 ± 10 (20–68)	9 M, 11F 12 M, 8F	Pain intensity < 4 on a 10 cm VAS	MTrP diagnosis was performed following the criteria described by Simons et al. [19] and by Gerwin et al. [89] FHP was noted both seated and standing.	Suboccipital	• Active MTrPs were only found in CTTH patients. • CTTH patients with active MTrPs reported greater headache intensity and frequency than those with latent. • A craniovertebral smaller angle was positively correlated with increased headache frequency and negatively correlated with headache duration.
Fernández-de-las-Peñas (2005) [64]	Examiner blinded to diagnosis	15 CCTH 15 ETTH 15 CTRLs	37 ± 16 38 ± 14 38 ± 14 Range not reported	5 M, 10F 4 M, 11F 5 M, 10F	CTTH: Pain intensity < 4 cm on a 10 cm VAS TTH: Interictally	MTrP diagnosis was performed following the criteria described by Simons et al. [19] and by Gerwin et al. [89]	Superior oblique	• 86% CTTH patients and 60% ETTH patients reported referred pain from MTrPs. • The pain was perceived as a deep ache located at the retro-orbital region – sometimes extending to the supraorbital region or the homo-lateral forehead. • Pain intensity was greater in CTTH patients than in ETTH patients.
Harden (2009) [73]	Double-blinded	23 CTTH with active cervical MTrPs (12 in active group, 11 in placebo group)	49.6 in active group 40.8 in placebo group Range not reported	7 M, 5F 7 M, 4F	Not reported	Patients received i.m. injections of botulinum toxin A or isotonic saline (placebo) in MTrPs. 25 units dose pr. MTrP, but no more than 100 units in total pr. patient (maximum four trigger points treated pr. patient).	Sternocleidomastoid, trapezius, and splenius capitis (which overlies involved cervical muscle groups: semispinalis capitis, longissimus capitis, recti capitis posterior and obliquus capitis superior)	• Patients in the active group reported greater reductions in headache frequency during the first part of the study, but these effects dissipated by week 12.
Karadas (2013) [74]	Double-blinded	48 CTTH with active MTrPs (24 in active group, 24 in placebo group).	40.4 ± 12 in active group 40.7 ± 13.2 in placebo group Range not reported	4 M, 20F 5 M, 19F	Not reported	Patients received i.m. injections with 0.5% lidocaine or 0.9% NaCl (placebo) to the trigger points of the muscles innervated by C1-C3 and the trigeminal nerve, exit point of the fifth cranial nerve and around the superior cervical ganglion.	Muscles innervated by C1-C3 and the trigeminal nerve, exit point of the fifth cranial nerve and around the superior cervical ganglion	• Patients in the active group reported significantly greater reductions in headache frequency and intensity.
Lattes (2009) [75]	None	27 CTTH	Approximately 46 (18–80)	7 M, 20F	Not reported	I.m. injections with gonyautoxin in 10 landmarks considered as MTrPs. EMG examination before and after injections.	Occipitalis and trapezius	• Responders (70%) had an average of 8,1 weeks free of pain following treatment. • The EMG recorded immediately after injection in all cases showed that the hyperactivity in the trapezius muscle was completely abolished.
Moraska (2017) [76]	Single-blind	34 CTTH 28 ETTH Massage: 13 CTTH 7 ETTH Placebo: 11 CTTH 10 ETTH Wait-list 10 CTTH 11ETTH	31.2 ± 11.3 34.4 ± 10.7 33.0 ± 9.0	7 M, 55F 1 M, 19F 2 M, 19F 4 M, 17F	Not reported	Individuals with ETTH or CTTH were randomized to receive 12 twice-weekly 45-min massage or sham ultrasound sessions or wait-list control. Massage focused on MTrPs. PPT was assessed using an algometer. MTrP diagnosis was performed following the criteria described by Simons et al. [19]	Suboccipital and upper trapezius	• PPT increased across the study timeframe in all four muscle sites tested for massage, but not sham ultrasound or wait-list groups.
Moraska (2015) [77]	Single-blind	30 CTTH 26 ETTH	32.1 ± 12 in active group 34.7 ± 11 in placebo group Range not reported	8 M, 48F (2 M, 15F in active group; 2 M, 17F in placebo group; 4 M, 16F in wait-list group)	Not reported	56 patients with TTH were randomized to receive 12 massage or placebo (detuned ultrasound) sessions over 6 weeks, or to wait-list. Massage focused on MTrPs in cervical musculature. PPT was assessed using an algometer. MTrP diagnosis was performed following the criteria described by Simons et al. [19]	Suboccipital, sternocleidomastoid, and upper trapezius	• Headache frequency fell in both the massage and the placebo group. • PPT improved in the massage group.
Palacios-Ceña (2016) [78]	Examiner blinded to diagnosis	77 CTTH 80 ETTH	46 (42–50) 47 (43–51)	46 M, 111F	Interictally	MTrP diagnosis was performed following the criteria described by Simons et al. [19] PPT was assessed over the trigeminal area, extra-trigeminal area and two distant pain free points using an algometer.	Temporalis, masseter, suboccipital, sternocleidomastoid, splenius capitis, and upper trapezius	• No difference in number of MTrPs and PPT in the two groups. • There was a significant negative correlation between the number of trigger points (active or latent) and PPT.
Romero-Morales (2017) [79]	None	60 ETTH 60 CTRLs	38,30 ± 10,05 34 ± 8,20 Range not reported	24 M, 32F 27 M, 33F	Not reported	MTrP diagnosis was performed following the criteria described by Simons et al. [19] PPT was assessed using an algometer.	Temporalis and upper trapezius	Minimum clinical differences in PPT between TTH and CTRLs were • Right upper trapezius; 0,85 kg/cm^2^ • Left upper trapezius; 0;76 kg/cm^2^ • Right temporalis; 0;16 kg/cm^2^ • Left temporals; 0,17 kg/cm^2^
Sohn (2012) [80]	Examiner blinded to diagnosis	23 CTTH 36 ETTH 42 CTRLs	53.43 ± 16.97 51.11 ± 14.42 51.69 ± 16.18 Range not reported	2 M, 21F 7 M, 29F 8 M, 34F	Headache intensity < 3 on a 10 cm VAS	MTrP diagnosis was performed following the criteria described by Simons et al. [19] and by Gerwin et al. [89] FHP was used to evaluate posture abnormalities. Measurement of neck mobility was used to evaluate mechanical abnormalities.	Temporalis, suboccipital, sternocleidomastoid, and upper trapezius	• The number of active MTrPs was significantly greater in CTTH subjects than in ETTH subjects. • The number of active MTrPs were correlated with the frequency and duration of headache. • No correlations were observed for FHP or neck mobility.

*CTTH* chronic tension-type headache, *ETTH* episodic tension-type headache, *CTRLs* healthy controls, *F* female, *M* male, *MTrP* myofascial trigger point, *EMG* electromyography, *PPT* pressure pain threshold, *FHP* frontal head position, *VAS* visual analog scale, *NRS* numeric rating scale

#### The occurrence of myofascial trigger points in tension-type headache

There is a high occurrence of active and latent MTrPs in patients with TTH [63–67, 69–72, 79] Active MTrPs are found almost only in TTH patients compared to controls [63, 65, 69, 72, 80]. MTrPs are more prevalent on the dominant side of the patients [66]. The number of active MTrPs is higher in adults in comparison to adolescents regardless of no significant association between the number of active MTrPs and headache frequency, duration and intensity [62]. Other studies have found that active MTrPs are correlated with the severity of TTH [65, 67, 78, 80] with a greater occurrence of MTrPs in chronic TTH in comparison to episodic TTH [80]. Furthermore, studies show that active MTrPs are correlated with the intensity, duration and frequency of headache episodes in TTH [65, 80]. In contrast, other studies failed to show a correlation between MTrPs and chronic and frequent episodic TTH [78] and showed no correlation between MTrPs and headache parameters either in episodic TTH patients [69].

#### Neck mobility and specific muscles

Episodic TTH patients had less neck mobility compared to controls [69]. Patients with active MTrPs had a greater forward head position than subjects only with latent MTrPs [69]. However, neither forward head position or neck mobility was correlated with headache parameters [69]. In a different study, active MTrPs in the right upper trapezius muscle and left sternocleidomastoid muscle was correlated with a greater headache intensity and duration [72]. Furthermore, active MTrPs in the right and left temporalis muscles correlated with longer headache duration and greater headache intensity, respectively [72]. Suboccipital active MTrPs correlated with increased intensity and frequency of headache [65]. Chronic TTH patients with active MTrPs in the analyzed muscles had a greater forward head position than those subjects only with latent MTrPs [65, 72]. Sohn et al. [80] identified a greater occurrence of MTrPs in chronic TTH compared to episodic TTH and that the number of active MTrPs correlated with the frequency and duration of headache, although they did not find any correlations for forward head posture and neck mobility in contrast to Fernández-de-las-Peñas et al. [65, 72].

#### Pressure pain threshold

The number of active and latent MTrPs was significantly and negatively associated with pressure pain thresholds on the temporalis muscle, C5/C6 zygapophyseal joint, second metacarpal, and tibialis anterior muscle [78]. Thus, a higher number was associated with a more generalized sensitization regardless of the frequency of headache. Another study observed that the location of active MTrPs in the temporalis muscle corresponded to areas with lower pain pressure thresholds which establishes a relationship between the two [68]. The same group found that chronic TTH patients with bilateral active MTrPs in the trapezius muscles have a significantly lower pain pressure threshold compared to patients with only unilateral active MTrPs [70]. Minimum clinical differences in pressure pain thresholds in TTH patients may be used to evaluate treatment of MTrPs [79].

#### Therapeutic studies targeting myofascial trigger points

Karadas et al. [81] investigated pericranial lidocaine injections in MTrPs in 108 patients with frequent episodic TTH using a double-blind placebo-controlled randomized study design. Repeated local lidocaine injections into the MTrPs in the pericranial muscles reduced both the frequency and intensity of pain compared to placebo. Another placebo-controlled study found similar results with lidocaine injections in MTrPs in chronic TTH with a reduction in pain frequency, pain intensity, and analgesic use [74]. In addition, there was a significant effect on anxiety and depression of the subjects. A randomized, double-blind, placebo-controlled pilot study of botulinum toxin A injections in MTrPs included 23 patients with chronic TTH [73]. The subjects were assessed at 2 weeks, 1, 2 and 3 months after injection. The botulinum toxin A group reported a reduction in headache frequency that disappeared by week 12. There was no difference in intensity between the groups. In a randomized, placebo-controlled clinical trial, Moraska et al. applied massage focused on MTrPs of patients with TTH [77]. For both active and placebo groups, there was a decrease in headache frequency, but not for intensity or duration. Thus, there was no difference between massage and placebo [81].

## Discussion

Ultrasound and EMG appear to be the most promising modalities to be used as a diagnostic test for MTrPs. While the use of ultrasound in headache disorders has primarily been focused on vascular changes and not on myofascial structures [82], ultrasound may also be used to identify MTrPs if specific analysis methods are applied [29] or with the use of elastography [27, 30, 32]. However, there is no precise description of a gold standard using these techniques, and they have yet to be evaluated in headache patients. Active MTrPs affect the electrical activity at rest and during muscle contraction in EMG studies [33–36]. Out of the two modalities, ultrasound is presumably the most viable candidate as a diagnostic test as it has an immediate availability at most treatment sites, it is time-efficient and is non-invasive. Although there are currently no studies investigating if it is possible to identify MTrPs with ultrasound without prior manual palpation. Future studies should investigate if ultrasound is comparable with manual palpation in identifying MTrPs. The other modalities do not appear to be suitable as microdialysis show mixed results regarding whether the local milieu of MTrPs is changed and needs further exploration before a conclusion can be made [25, 26, 83]. According to the review by Dibai-Filho et al. [37], infrared thermography appears to be a promising non-invasive method, but it should still only be used as an auxiliary tool in the evaluation of MTrPs due to conflicting results. Magnetic resonance elastography in diagnosing MTrPs has only been investigated in a few studies, and the sensitivity may be too low for suitable use as a diagnostic test [42].

**Fig. 1:**
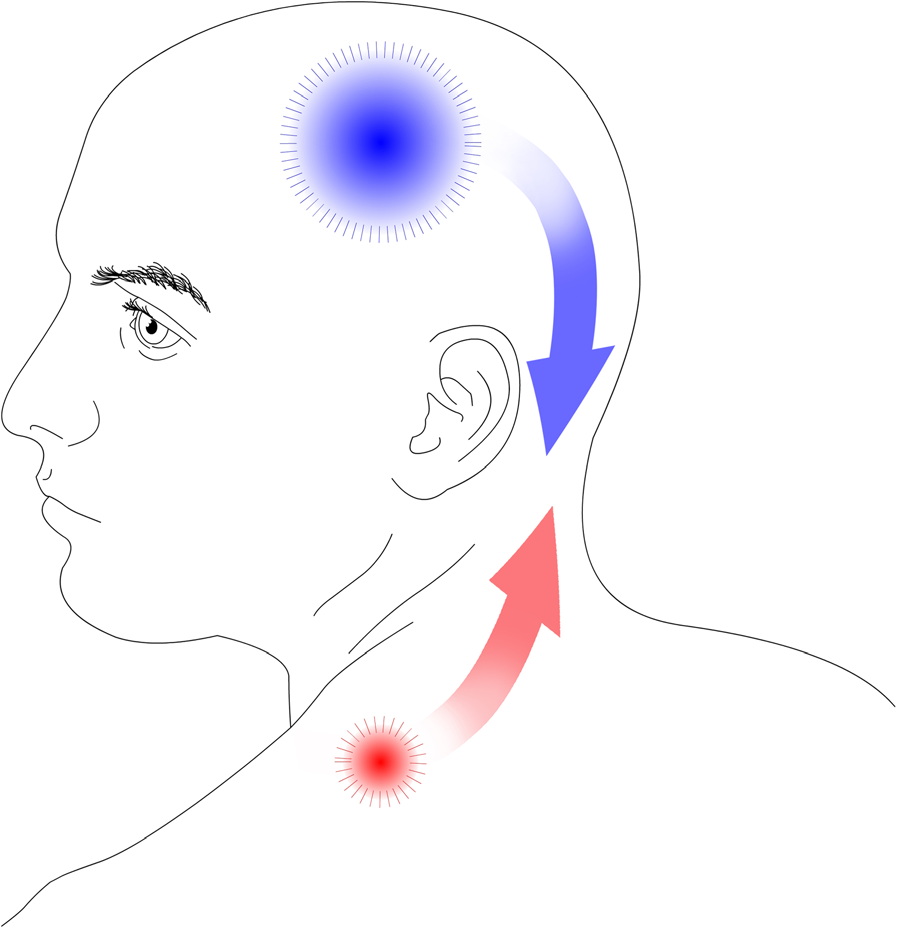
The bottom-up model states that increased peripheral nociceptive transmission sensitizes the central nervous system to lower the threshold for perceiving pain while the top-down model suggests these changes are already present in central nervous system. In relation to myofascial trigger points, a bottom-up model would suggest that increased nociceptive transmission from myofascial trigger points lowers the threshold for perceiving pain (red). A top-down model would suggest that central sensitization may contribute to the occurrence of myofascial trigger points rather than the other way around (blue)

Studies show a high occurrence of active and latent MTrPs [45–49] and correlation between neck mobility and MTrPs in migraine patients [46, 48, 49, 58]. However, there are conflicting results in which muscles are the most affected [47, 48], and it is unclear whether there is a positive correlation with the degree of headache frequency or intensity due to conflicting results. Palpation of MTrPs may provoke a migraine attack in some patients [45, 53] but needs further confirmation in placebo-controlled studies. Intervention studies targeting MTrPs are mostly positive [50–52, 56, 57], but they lack placebo-control. Thus, a bottom-up association between MTrPS and migraine [44] cannot be fully supported based on the evidence (Fig. 1). In addition, in patients with migraine-fibromyalgia comorbidity, it has been shown that migraine attacks exacerbate fibromyalgia symptoms, suggesting a top-down central sensitization [84] as fibromyalgia symptoms include specific tender points [85]. Although a study showed migraine severity was similar in migraine patients with and without fibromyalgia [86]. One would expect an association between migraine severity and co-existing fibromyalgia if a top-down central is taking place in patients with this comorbidity. It is possible that MTrPs may have an important role in some subpopulations of migraine patients. This calls for therapeutic studies targeting patients with a high degree of MTrPs, but this is only speculative at this point.

The prevalence of active MTrPs in TTH [65–67, 73, 74, 77, 78, 80, 81] is coherent with the hypothesis that peripheral mechanisms are involved in the pathophysiology of TTH [14–16, 60]. It has been speculated that an increased peripheral nociception increases the sensitization of central mechanisms resulting in an increase in the sensitivity to peripheral pain (Fig. 1). Active MTrPs may contribute to a central sensitization as they are correlated with lower pain pressure thresholds [68, 70, 78]. This would also provide an explanation for the efficacy of injections of lidocaine in MTrPs [74, 81] as these would reduce the transmission of peripheral nociception. However, these assumptions are in contrast with a study showing that the number of active MTrPs is higher in adults in comparison to adolescents, regardless of no significant association with headache parameters [62]. This suggests that active MTrPs are accumulated over time as a consequence of TTH [62] instead of being an integrated part of the pathophysiology of TTH. Previous studies of botulinum toxin A injections in pericranial muscles have been shown to have no effect in TTH [87]. The efficacy of botulinum toxin A in MTrPs [73] might be explained by its possible action of modulating the release of nociceptive and inflammatory mediators e.g., CGRP and SP [88]. These inflammatory mediators may be increased in the local milieu of MTrPs [25, 26]. This would also account for its poor efficacy in injection protocols targeting fixed landmarks in pericranial muscles instead of MTrPs [87], as these substances appear to be concentrated at MTrPs [25, 26].

There are many overlapping findings in studies of MTrPs in migraine or TTH. In both disorders, MTrPs are prevalent and may be related to neck mobility. Palpation of MTrPs can, in some cases, provoke an attack in migraine patients, while palpation of MTrPs in TTH can provoke pain resembling the usual headache pattern of patients. Intervention studies are promising in both disorders. The quality of studies in both disorders varies greatly as many of the reviewed studies lacked blinding (Table 3). Furthermore, true blinding is difficult to achieve as active MTrPs by definition cause referred pain.

**Table 3:** An overview on the use of blinding, control groups and placebo

	Migraine	Tension-type headache	Total
Blinding	36% (5/14 relevant studies)	79% (15/19 relevant studies)	61% (19/33 relevant studies)
Control group	44% (4/9 relevant studies)	79% (11/14 relevant studies)	65% (15/23 relevant studies)
Placebo	40% (2/5 relevant studies)	80% (4/5 relevant studies)	60% (6/10 relevant studies)

## Conclusion

In conclusion, ultrasound elastography is the most promising tool to assess MTrPs [27, 30, 32], but still needs to be performed combined with palpation, which introduces risk of bias and interobserver variation. MTrPs are very frequent in both migraine patients [45–49] and TTH patients [65–67, 73, 74, 77, 78, 80, 81] compared to healthy controls. Active MTrPs are especially interesting as these are rarely found in control groups. However, their role in the pathophysiology of each disorder and to which degree is still unclear. The results of the provocation and intervention studies support the hypothesis of a trigemino-cervical-complex pathophysiology model in both migraine [45, 50–53, 56, 57] and TTH [73, 74, 81]. Whether MTrPs contribute to an increased disease burden in migraine is uncertain [45, 47, 48] and needs further exploration [50, 52]. Future research should aim to increase the quality of studies before further speculations are made. To elucidate this, large-scale studies to stratify the headache populations into more homogenous subgroups should be conducted.

## Abbreviations

BK: Bradykinin
CGRP: Calcitonin gene-related peptide
CTRL: Healthy control
EMG: Electromyography
F: Female
FHP: Forward head posture
IL-1β: Interleukin 1 beta
IL-6: Interleukin 6
IL-8: Interleukin 8
M: Male
MA: Migraine with aura
MO: Migraine without aura
MTrP: Myofascial trigger point
NE: Norepinephrine
NRS: Numeric rating scale
PPT: Pressure pain threshold
SP: Substance P
TNF-α: Tumor necrosis factor alpha
TTH: Tension-type headache
VAS: Visual analog scale
